# Iron in Functional Nervous Disorders

**Published:** 1898-02

**Authors:** S. G. Courtney Pinckney

**Affiliations:** Atlanta, Ga.; Lecturer on Diseases of the Mind and Nervous System, Southern Medical College


### Southern Medical Record.

A MONTHLY JOURNAL OF MEDICINE AND SURGERY.

VOL. XXVIII. ATLANTA, GA., FEB. 1898. NO. 2

### Original Articles.

### IRON IN FUNCTIONAL NERVOUS DISORDERS.

S. G. COURTNEY PINCKNEY, M. D., Atlanta, Ga.

Lecturer on Diseases of the Mind and Nervous System, Southern Medical College.

Anæmia is a very constant condition in hysteria and neurasthenia; often the result, it is more frequently the determining cause of the attack. In the emaciated and feeble its presence is immediately noted, while in the stout and apparently physically robust it is frequently unsuspected.

A careful examination of the blood will show that even in this last class the hemæglobin is markedly diminished as a rule. Stout and well-nourished as they appear they are constantly in a state of physical exhaustion with a feeble muscular system and lowered resisting power, padded out with loosely packed masses of physiologically inert fat—fat showing no tendency to undergo proper metabolism—they are anything rather than the healthy people they seem to be.

It is in this type that treatment is so frequently unsatisfactory, principally because the physical deterioration is apt to be overlooked. Routine rest and feeding is rarely used, as it is in the palpably asthenic cases. The tendency seems to be to regard them more as lithæmics than neurasthenics, and exercise and abstemiousness are unduly insisted upon.

It is in this very class of patients so resistant to most methods of treatment that iron gives us brilliant results; in fact iron with a mild saline water in the morning is generally quite sufficient to effect a cure. Even modification of mode of life is rarely necessary, though massage is a very valuable adjunct giving us all the good effects we look for and so rarely see in the thin and exhausted cases. That characteristic condition aptly called "plethora" is certainly a rare one in this age; its modern prototype "hyperæmia" is in reality an anæmia.

It is not sufficient to simply replace the lost hemæglobin—iron should be kept up (in diminished doses) for at least a month after the hemæglobin percentage is normal. We are apt to lose sight of its other good effects if we only think of it in relation to the blood count. Under its use the appetite is rapidly increased, the heart's action becomes stronger, sleep and digestion are better and mental weariness is lessened. As these symptoms are due to malnutrition, we would expect their disappearance with an improvement in the quality of the blood; it is a clinical fact, however, that their improvement appears long before there could be any appreciable change in the blood—often in the first or second day. It is rarely necessary to use any other stimulants of appetite or digestion, and neither necessary or advisable to advise digestive ferments or prepared foods.

I have had good success with Dialized Iron, and with Sulphate of Iron (in form of Blaud's pills), but I prefer Gude's Pepto-Mangan to either. The disagreeable effects of iron are the head symptoms and constipation. Head symptoms are rarely, if ever, present while using Pepto-Mangan, and the constipation is much less than with other preparations often only lasting for the first few days.

The commercial Blaud pill has about the consistency of a cobble stone, probably about its virtues as a remedy. If Blaud's formula is desired it should be put up fresh and dry in capsule form. The efficiency of iron is increased by a saline before breakfast—preferably Sulphate of Soda in a full glass of hot water. Several quarts of any pure water drunk through the day is of distinct advantage.

